# Global impact of COVID-19 on corneal donor tissue harvesting and corneal transplantation

**DOI:** 10.3389/fmed.2023.1210293

**Published:** 2023-08-07

**Authors:** Morteza Mousavi, Nicolás Kahuam-López, Alfonso Iovieno, Sonia N. Yeung

**Affiliations:** Department of Ophthalmology and Visual Sciences, University of British Columbia, Vancouver, BC, Canada

**Keywords:** COVID-19, eye bank, corneal transplant, donor tissue, tissue procurement and processing

## Abstract

**Introduction:**

The purpose of this review is to consolidate and examine the available literature on the coronavirus disease 2019 pandemic and its effect on corneal transplantation and eye banking.

**Methods:**

A primary literature search was conducted using the PubMed (Medline) database with keywords and MeSH terms such as “corneal transplantation,” “eye banks,” “keratoplasty” and then were combined with COVID-19. Relevant articles through September 2022 were assessed and 25 articles were included in this review.

**Results:**

Donor tissue volumes declined globally during lockdown periods due to a lower number of referrals and tighter tissue screening guidelines. Rates of elective surgeries decreased in the lockdown period compared to respective periods in previous years. However, changes in rates of emergency procedures were not uniform across different regions. Moreover, rates of different elective corneal grafts [i.e., penetrating keratoplasty (PK), endothelial keratoplasty (EK), or anterior lamellar keratoplasty (ALK)] were affected differently with the pattern of change being dependent on region-specific factors.

**Conclusion:**

Both donor tissue volumes and rates of corneal transplant procedures were affected by lockdown restrictions. The underlying etiology of these changes differed by region. Examining the range of impact across many countries as well as the contributing factors involved will provide guidance for future global pandemics.

## 1. Introduction

In late 2019, the emergence of the severe acute respiratory syndrome coronavirus 2 (SARS-CoV-2) sparked the beginning of the coronavirus disease 2019 (COVID-19) pandemic. There have been over 450 million confirmed cases and over 6 million confirmed deaths from COVID-19 (1). As countries around the globe were grappling with the fast spread of the disease, several restrictions were put into place to help reduce the growing number of cases. One such restriction was the cancellation of many elective procedures (2, 3). As a result, organ and tissue transplant volumes decreased during the first months of the pandemic (4). This included corneal transplants, as the risk of transmission through ocular tissue transplantation was and still is a matter of contention (5). Given these challenges and uncertainties, many eye banks experienced decreased donor tissue availability and subsequently, the number of corneal transplantation procedures declined. Based on the statistical report published by the Eye Bank Association of America (EBAA), transplant volumes in 2021 recovered from the decline in 2020. However, the numbers have yet to match the 2019 data (6). Therefore, understanding the differential challenges and responses to the pandemic in different regions is instrumental in planning ahead for similar scenarios in the future. Herein we examine reports of eye banking and corneal transplant statistics during the COVID-19 pandemic and explore the reasons behind the region-specific impact.

## 2. Materials and methods

**Figure 1:**
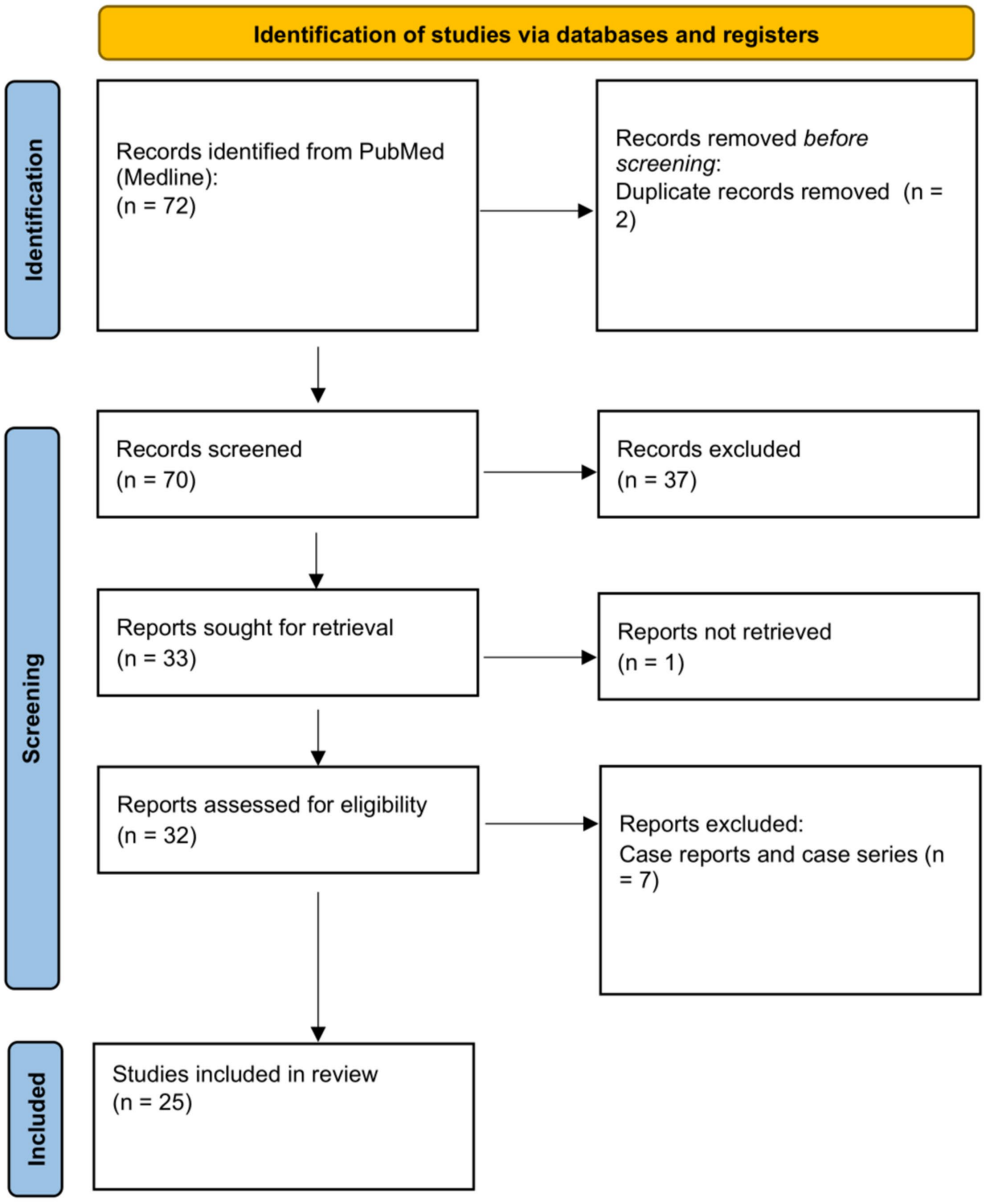
PRISMA flow diagram.

A primary literature search was conducted in November 2022 using the PubMed (Medline) database with keywords and MeSH terms such as “corneal transplantation,” “eye banks,” “keratoplasty,” and “COVID-19.” There were no publication year or language restrictions. The search yielded a total of 72 articles (Figure 1). Case reports and case series were excluded from the results. Abstracts were reviewed, and literature reporting statistical data on corneal tissue donor volumes and transplantation during the pandemic were selected. Secondary literature was found using pertinent references from the primary articles. A total of 25 relevant papers were identified and reviewed.

## 3. Results and discussion

### 3.1. Ocular donor tissue volumes

Studies from the United States (7–9), United Kingdom (10), Canada (11), Germany (12), Italy (13–15), India (16–20), and continental data from Europe (21) showed a decrease in the number of donor corneal tissues procured during their respective lockdown periods in 2020. However, the magnitude of this decline varied among the different regions (Table 1). Interestingly, one survey of members of the European Eye Bank Association (EEBA) found that only one country (Bulgaria) of 19 participating countries showed an increase in the number of tissues procured and distributed from March to May 2020 compared to the same period in 2019. However, this was attributed to the small number of corneas (only 28) procured in this region during and before the period of interest (21).

**Table 1:** Studies comparing the number of procured tissues between the lockdown periods in 2020 and respective months in 2019.

Study	Region	Comparison period	No. of procured tissue in 2020	No. of procured tissue in 2019	% decline	*p*
Ballouz et al. (7)	Eversight eye bank facilities (Michigan, Ohio, Illinois, New Jersey, And Connecticut)	March–June	Not reported	Not reported	45	0.031
AlShaker et al. (11)	Eye bank of Canada (Ontario Division)	March–June	267	769	65	Not reported
Trigaux et al. (12)	26-member eye banks of the German Ophthalmological Society	March–April	1,453	1,758	17	Not reported
Aiello et al. (13)	13-member eye banks of the Italian Society of Eye Banks	March–April	1,284	3,088	58	< 0.0001
Parekh et al. (14)	Fondazione Banca degli Occhi del Veneto (FBOV – Venice, Italy)	March–April	Not reported	Not reported	41	< 0.0001
Agarwal et al. (18)	Apex health institute of India	March–July	Not reported	Not reported	99	<0.001
Nathawat et al. (17)	Eye Banks and Cornea Surgeons’ members of the All India Ophthalmological Society (AIOS) and the Eye Bank Association of India (EBAI)	March–May	1,898	8,735	78	Not reported

Several reasons have been reported in the literature to explain this decrease in donor tissue volumes. AlShaker et al. (11) and Ballouz et al. (7) interpreted the decrease in the number of eligible referrals to their respective eye bank as a reflection of an increased number of COVID-19 cases as well as more stringent screening criteria for tissue selection. Moreover, the rate of conversion of those eligible referrals to retrieved tissues for transplantation decreased in the same period in 2020 compared to 2019. This change in conversion rates was thought to reflect logistical challenges due to COVID-19 such as lack of staffing (11). Issues with staffing were also reflected in Thuret et al. (21) survey of EEBA members. The issue of logistical challenges was also highlighted in a survey of eye banks in Germany, where 21 out of the 26 surveyed eye banks reported a decrease in their activity since the beginning of the pandemic compared to before the pandemic (12).

Although donor screening criteria was often reported to account for the decrease in donor tissue volumes, these criteria were variable. A survey of 64 eye bank members of the EEBA reported by Thuret et al. (21) showed that all eye banks in their study contraindicated donations from patients whose confirmed cause of death was COVID-19. However, screening criteria for the rest of the donors, for example, those who had COVID-19 but died of other causes or those who had recovered from a COVID-19 infection and then passed away from other causes later on, varied among the different eye banks. In their survey, the least stringent criteria mandated a 14-day symptom-free period before death, whereas the most stringent criteria mandated a period double that time. Moreover, they reported that the definition of COVID-19 symptoms varied among different exclusion guidelines, with the strictest criteria mandating exclusion of those with “unexplained cough, unexplained asthenia and myalgia, intermittent fever, shortness of breath or unexplained conjunctivitis before death.” Such variability in screening criteria was also observed in a survey of Indian eye banks (17), which reported that a fraction of the eye banks avoided tissue retrieval from COVID-19 positive cases (44.4%) or those with suspicious respiratory symptoms (36.7%). In contrast, some eye banks (16.67%) completely halted tissue collection. A survey of German eye banks (12) showed all active banks followed local guidelines. At the same time, a portion of them also followed recommendations made by the Global Alliance of Eye Bank Associations (GAEBA) or the European Center for Disease Prevention and Control (ECDC). The variability in screening criteria is also reflected in the differences in the reported rate of tissue exclusion due to COVID-19 in different studies, which varied from a 2-month rate of 2% in the Italian Society of Eye Banks (SIBO) reports (13) to 35% reported for the month of April in Eye Bank of Canada Ontario Division’s (EBCOD) analysis (11). Thuret et al. (21) did establish that higher levels of stringency in the screening criteria often led to a decrease in tissue procurement. This diversity in donor screening criteria not only reflects adherence to the guidelines set forth by different regulatory bodies but is also a product of the timing of when these guidelines were adopted. Furthermore, as our knowledge of SARS-COV2 evolves with time, the guidelines may also be refined to reflect new data (22).

Numbers of procured tissues from the SIBO and Fondazione Banca degli Occhi del Veneto (FBOV eye bank—Venice, Italy) showed some improvement in the first month following lockdown but not large enough to statistically match the comparison periods in previous years (13, 14). A survey conducted by the EBAA showed a similar pattern of improvement in the tissue distribution volume 2 months after lockdown (8). In a study of the Eversight eye bank, Ballouz et al. (9) compared the number of available corneal tissues and corneal transplant surgeries in an 18-month period from July 2020 to December 2021 (when elective surgeries were resumed) to the same period in 2018 and 2019. The number of surgeries requiring corneal tissue significantly increased in this period compared to the pre-pandemic period, and the number of suitable tissues was similar in both periods of comparison. Therefore, though the number of procured tissue increased during this period it was not enough to meet the needs due to the increased demand. This shortage in tissue was handled through an increase in imported tissues (9). Ongoing efforts to improve the rates of donation of corneal tissue post-pandemic will continue to address the increasing demand for corneal transplantation worldwide.

Lastly, regarding the number of exported tissues the evidence seems to be contradictory. The data from the Eversight eye bank shows a significant decrease in exported tissues which is congruent with the national decline reported by EBAA (7, 8). On the other hand, Aiello et al. (13) reported an increase in the number of exported tissues from SIBO. They attributed this rise to the fact that the lockdown started in Italy before other European countries, and at that time, exports were still happening to those countries where elective surgeries had not stopped (13).

### 3.2. Transplant volumes

In general, the number of ophthalmic procedures significantly decreased during the lockdown phase in many regions of the world (18, 23–26). Analogous to donor tissue volumes, a decrease in the volume of transplanted tissues and the number of procedures completed during the first wave of the pandemic is reported in the literature (7, 11, 13, 19, 20, 27–30). Similar to the change in donor tissue volumes, the reported decline in transplant volumes varied among regions (Table 2). This was a consequence of non-emergent procedures being shut down due to special considerations in resource reallocation. We will explore these findings in more detail below.

**Table 2:** Studies comparing the number of surgical procedures between the lockdown periods in 2020 and respective months in 2019.

Study	Region	Comparison period	No. of Surgical procedures in 2020	No. of surgical procedures in 2019	% decline	*p*
Aiello et al. (13)	13-member eye banks of the Italian Society of Eye Banks (SIBO)	March–April	534	1,220	56	<0.0001
AlShaker et al. (11)	Eye bank of Canada Ontario Division (EBCOD)	March–June	207	753	73	Not reported
Ballouz et al. (7)	Eversight eye bank facilities (Michigan, Ohio, Illinois, New Jersey, and Connecticut)	March–June	Not reported	Not reported	53	0.011
Din et al. (29)	Moorfields Eye Hospital (London, UK)	April–June	10	163	92	Not reported

Because mainly elective procedures were subject to cancellations during COVID-19 lockdown periods, the number of emergency procedures would not be expected to change compared to pre-pandemic years (13, 29, 31). Interestingly, Din et al. (29) reported an increase in the number of emergency procedures due to sharp object trauma (29), which was attributed to redirection of all ophthalmic services from other centers to their hospital as well as an increase in domestic incidences due to the lockdown (29). On the other hand, dell’Omo et al. (26) reported a significant decrease in the number of elective and emergency procedures compared to the same period in 2019 in six centers in Italy. Moreover, a 2-month analysis of 39 centers in Italy showed a significant decrease in some subtypes of emergency procedures compared to the same period in 2019 (24), which was reported to be a result of the limitations in access to operating rooms as their availability was reduced in all except one center.

**Figure 2:**
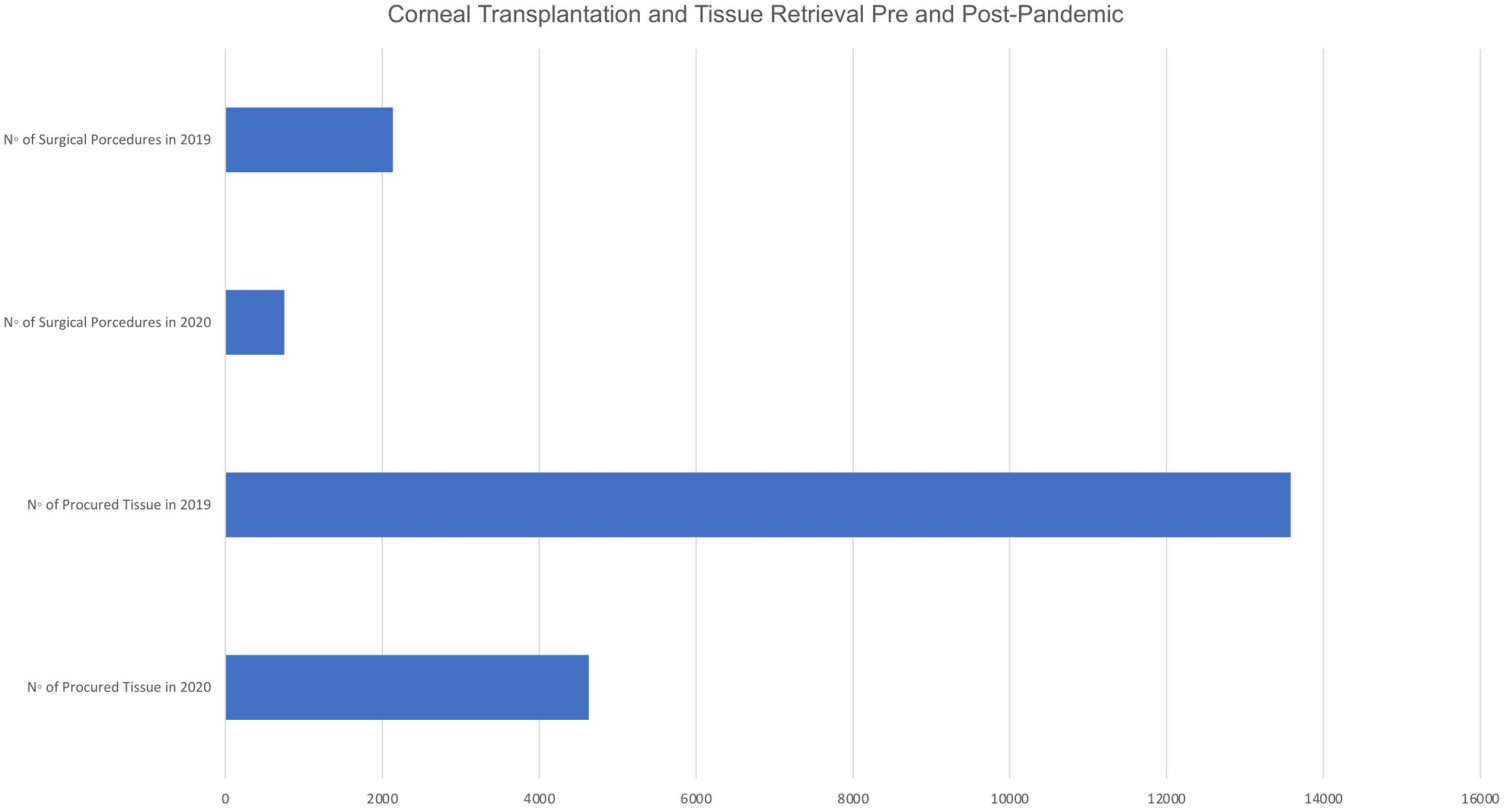
Corneal transplantation and tissue retrieval pre- and post-pandemic.

Descriptive statistics drawn from many eye banks around the world showed a decline in transplanted tissue volumes in 2020 compared to 2019 (Figure 2) (7, 11, 13, 20). Specifically, a decline both in donor tissue and transplant volumes was noted. A drop in the rate of unused tissue during the lockdown period compared to the yearly average in 2019 was reflected in the EBCOD records (11). This was specifically recorded in the months of April and May, when only emergency procedures were done. As a result, a small number of tissues was released for transplant and utilization of retrieved tissues was maximized. However, they reported a surplus of unused tissue in March when operating rooms (ORs) were closed and a return to rates similar to 2019 in the month of June when ORs resumed regular activity. Das et al. (20) reported an increase in the average rate of corneal tissue utilization during and after COVID-19 lockdown in 2020 compared to the same average rate in 2019 due to a shortage of donor tissue availability post-lockdown. As a result, the stringency of their selection criteria was reviewed to improve utilization.

On the other hand, other centers showed a surplus of corneal tissue during the lockdown period due to the reduction in scheduled surgeries and a significant rise in the number of canceled transplants (7, 13). Due to an increase in the number of wasted donor tissues, Busin et al. (27) underlined the importance of exploring alternative procurement techniques that would extend the storage time of procured corneal tissues and reported tissue dehydration as a useful alternative method, especially in the case of deep anterior lamellar keratoplasties. A survey of eye banks and surgeons in India showed a statistically significant increase in the use of long-term tissue preservation methods in the same period in 2020 compared to previous years (17).

When examining the distribution of different types of corneal transplantation surgeries, there was no trend noted. By analyzing Brazilian national data and records from the state of São Paulo, Moriyama et al. (28) found that the proportions of tectonic and/or therapeutic procedures significantly increased. Conversely, the proportions of all 3 optical procedures [penetrating keratoplasty (PK), endothelial keratoplasty (EK), or anterior lamellar keratoplasty (ALK)] significantly decreased, with EK and especially ALK showing more dramatic declines than PK. The analysis of the same proportions after removing the moratorium showed that while the proportions of all 3 optical procedures increased, PK rates showed a significant increase compared to the rates in 2019, whereas EK rates were still below those of 2019 (28). One explanation the authors suggest to account for the change in EK proportions is that EKs are mostly done on elderly patients and during the moratorium as well as after it was released the older population was facing stricter mobility restrictions. The United Kingdom (UK) data did not show any meaningful changes in the patterns of corneal transplants from April 2020 to March 2021 compared to similar periods in previous years (10). The EBAA data revealed a decrease in EK proportions and an increase in PK proportions during the lockdown in the US, however the proportions were once again similar to pre-pandemic values after the restrictions were lifted (28). Mencucci et al. (15) showed a decrease in the rates of both PKs and ALKs due to the suspension of elective surgeries. PKs showed a steeper decline, explained by the general trend of preferring ALKs over PKs in recent years. The increase in the rates of EKs during the lockdown period was attributed to the use of local anesthesia during EKs. In their analysis of data from a tertiary eye care center in India, Das et al. (20) reported a decline in the numbers of PK, EK, and ALK procedures, but a marginal increase in the number of therapeutic penetrating keratoplasty (ThPK) compared to pre-lockdown data (20) due to advanced worsening of visual acuity in patients with infectious keratitis during the pandemic (20).

Data from several eye banks showed an increase in the number of procedures done after the lockdown period, however, those post lockdown numbers were still significantly lower than pre-pandemic rates (13, 20, 28).

### 3.3. Patients’ challenges

Several studies have analyzed data regarding logistical challenges facing patients requiring corneal transplants.

Data from Moorfields Eye Hospital (MEH) during the lockdown period showed a significant increase in the time between patient’s symptom presentation and surgical procedure compared to pre-pandemic period (29). The authors also examined patients’ travel distances during the lockdown and found an increasing trend compared to the year before the pandemic. Together, these two results highlighted the fact that during the pandemic corneal procedures were being done in only one of the several centers of the National Health Services (NHS) trust center in London while other centers had been redeployed for COVID-19 care. A geographical barrier was also reported by Das et al. (20). Their analysis of a tertiary eye care center in India showed a decreasing trend in the number of patients who travelled interstate to consult for corneal procedures.

Lastly, in their analysis of the national Brazilian data, Moriyama et al. (28) found a significant decrease in the number of new patients added to corneal surgery waiting list during the lockdown phase when compared to the same period in 2019. The authors suggested this was due to a decrease in the activity of outpatient clinics in that period.

## 4. Conclusion

This review summarizes the impact of COVID-19 on corneal donor tissue harvesting and corneal transplant in different settings around the world. Both donor tissue and transplant volumes showed a decline during the lockdown times. Moreover, geographical challenges were highlighted which resulted in longer distances traveled by patients to obtain care, as well as delayed care. Eye bank operations during the pandemic have been evolving due to knowledge gained from COVID-19 and the subsequent changes in guidelines over time. Region-specific variability in lockdown timing also affected downstream donor tissue criteria evolution and tissue availability for corneal transplantation. Understanding the impact of this pandemic on eye bank operations around the globe will ultimately allow us to anticipate the disruption to the delivery of corneal tissue for transplantation in future pandemics, and to address the impact more efficiently with the goal of resuming care for our patients. Reports from many regions are valuable to elucidate the region-specific factors with critical roles in providing safe high-quality corneal tissue, optimize eye bank guidelines, improve the efficient use of available tissue, and minimize tissue waste in future similar scenarios.

## Author contributions

MM, NK-L, AI, and SY participated in the idea conception, acquisition and data analysis, drafting of the manuscript, and final approval of the version to be published. All authors agree to be accountable for all aspects of the work in ensuring that questions related to the accuracy or integrity of any part of the work are appropriately investigated and resolved.

## Conflict of interest

The authors declare that the research was conducted in the absence of any commercial or financial relationships that could be construed as a potential conflict of interest.

## Publisher’s note

All claims expressed in this article are solely those of the authors and do not necessarily represent those of their affiliated organizations, or those of the publisher, the editors and the reviewers. Any product that may be evaluated in this article, or claim that may be made by its manufacturer, is not guaranteed or endorsed by the publisher.
